# Analysis of Color Consistency in Retinal Fundus Photography: Application of Color Management and Development of an Eye Model Standard

**DOI:** 10.1155/2014/398462

**Published:** 2014-12-30

**Authors:** Christye P. Sisson, Susan Farnand, Mark Fairchild, Bill Fischer

**Affiliations:** ^1^Rochester Institute of Technology, Rochester, NY 14623, USA; ^2^University of Rochester Medical Center, Flaum Eye Institute, Rochester, NY 14642, USA

## Background

Color variation in retinal fundus photography represents a significant gap in the standardization of color for fundus cameras. Fundus cameras are used in the context of ophthalmology as a method of documenting a patient's retina to monitor pathology over time. This form of ophthalmic imaging is also used in clinical trial research and, increasingly, teleophthalmology, as a stand in for an in-person examination. Given the increased reliance on these images as representations of the appearance of a patient's eye, it becomes important to identify inconsistencies between devices and provide the most accurate rendering of the retina possible.

**Figure 1:**
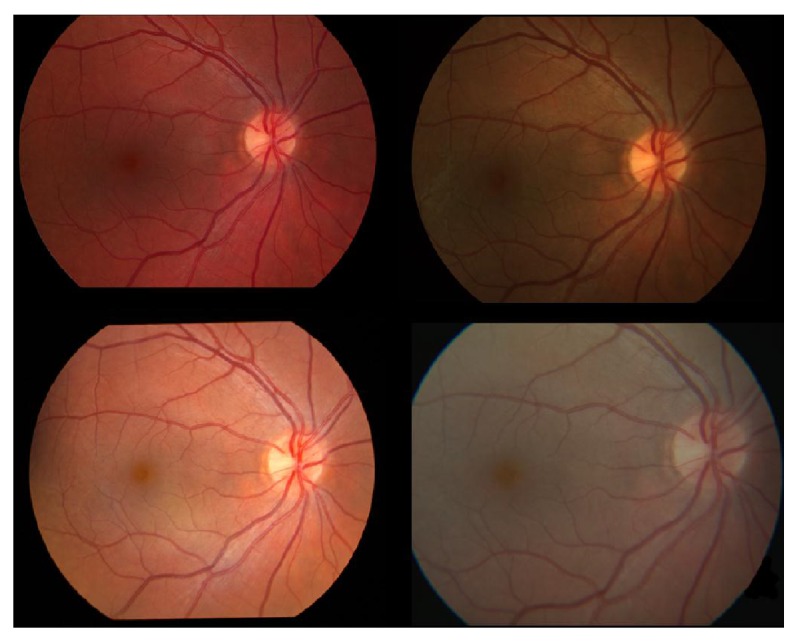
Examples of the variation of color among fundus images of the same retina.

This research aims to identify these inconsistencies and reconcile them by proposing an eye color model standard. The authors could not identify other attempts to reconcile retinal color at capture, only after the fact image adjustment [1, 2] or application of current color management practices (Figure 1) [3].

## Method

The method for both documenting and reconciling the color inconsistency in fundus cameras included photographing a standardized color target (ColorGauge Nano Target), inside a model eye with a standardized imaging protocol. Fundus cameras operate by projecting a donut of light from the lens through a dilated pupil to illuminate the retina. The model eye was utilized to mimic the imaging conditions of the eye as the other half of the optical system. After capturing on several fundus camera systems, the images were exported as TIFFs and compared to the original color targets. Colorimetric profiles for each camera were generated and applied back to the images to determine how closely the images could be made to match each other.

## Results

**Figure 2:**
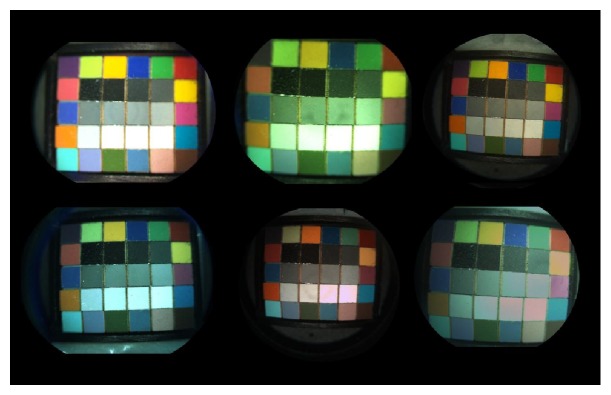
Six of the tested cameras test target images.

The testing showed significant color variation among 8 tested cameras, even among those from the same manufacturer. The resulting targets also demonstrated significant variation in contrast among the cameras tested. This is not unexpected given the cameras variety in terms of bit depth and sensor type, but we have not determined if these variations are products of the hardware and not part of the color and gamma correction applied at the imaging system software level by the manufacturer (Figure 2).

The application of individually generated camera profiles brought the color variations into much greater levels of agreement in the majority of cameras tested. Cameras that “failed” most often included those that had very high contrast, demonstrated by the greyscale patches in the target. Consequently, the color data in these images was much more limited, making it more difficult to bring the color into agreement with the original target.

## Conclusion

The testing confirmed the wide disparity in color consistency and accuracy among fundus camera systems. The color variations between the tested cameras were successfully mitigated by the application of the profile to the final images in most cases. However, the question remains if the color variation exists in the hardware of the cameras or in the image modifications that occur at the software level or both. In either case, the testing demonstrates that it is possible to bring cameras from a wide variety of manufacturers within greater color agreement than was previously displayed.
